# Are the Mineral Acids Formed in the Mouth?

**Published:** 1870-10

**Authors:** S. P. Cutler


THE
DENTAL REGISTER.

Vol. XXIV.] OCTOBER, 1870. [No. 10.

### Original Communications.

### "ARE THE MINERAL ACIDS FORMED IN THE MOUTH?"

BY S. P. CUTLER, M. D., D. D. S.

The above is the heading of an article in the May number of the *Missouri Dental Journal*, 1870.

All publications in the journals are regarded as public property and subject to criticism.

There are some assumptions in the above article, perhaps, not founded on sufficient data to warrant belief, and some of the chemical formulas not correctly stated. In the first place I will inquire, what constitutes a mineral acid in contra-distinction to any other acid. Sulphuric acid might with propriety be considered a true mineral acid, as one of its elements, sulphur, may be regarded a mineral element, the other element not, though an active agent or element in most acids only a few exceptions, its salts of the metals are minerals. Nitric acid is generally considered a mineral acid, though composed of two gases, neither of which could be regarded as a mineral element *per se*, neither is the acid itself uncombined mineral in character, it being a liquid under all circumstances when uncombined; but on the other hand, when combined with metallic oxides, they are strictly mineral in their character.

This acid may be regarded in one sense as of organic origin, it being formed by animal and vegetable putrefaction, fermentation by oxydation of ammonia, which is first formed in the presence of eight equivalents of nascent oxygen, the ammonia also being nascent; at the same time three equivalents of water are formed in the presence of potash, nitrate of potash is formed; nitric acid is also formed in thunder clouds, which was synthetically proven by Cavendish in 1780. These are the only known sources of nitric acid.

Hydrochloric acid or muriatic acid, also regarded as a mineral acid, is composed of two gaseous bodies, neither of which could be regarded as mineral, the acid being a liquid, its salts of the metals are regarded as minerals, more especially the halogens.

This acid is made by combustion of hydrogen in chlorine in a rapid manner and in a more gradual manner by slow chloridation of hydrogen, or by decomposition of chlorides already formed, which are minerals. Some acids are solids; others are liquid, and others gaseous.

The elements of all the organic acids are found in the mineral acids, namely: carbon, oxygen, and hydrogen; which are found in all organic acids, with one exception, that of oxalic, which has no hydrogen.

There are a certain five elements found to exist in organisms which are regarded as organic elements when so existing only. There are a still greater number of simple elements that are never found in organic life, called inorganic, not all of them mineral in their character. Some are metals, others mineral or metalloids, others gaseous. Now all the elements found in the mineral acids are found in organisms in greater or less quantity, without any exception.

The writer of the article entertains the idea that the mineral acids may be formed in the mouth, and gives his formulas for the same, based upon the supposed knowledge of the exact specific action of each individual acid on the teeth in the mouth. Whether or not the writer experimented with teeth out of the mouth, with the different acids named, he does not state. This would be the proper plan in making up his differential diagnosis of decays or action of acids on the teeth.

Now there are three different and distinct forms of Dental caries, the black, the brown, and the white, the first being characterized by disintegration of both dentine and dontine, or dentine and cartilage *pari passu*, leaving a discolored surface below, frequently hard and dense from the fact that this is the slowest of all forms of decay, hence more time being allowed for tubuli absorption for a short distance into the dentine below the decay, of coloring matters, and not from any specific acid action differing from either of the others; if so, I have not yet had convincing proof of the fact.

The brown variety is characterized by a removal of the lime salts down to an indefinite distance below or into the cartilage. In such cases the lime gives way faster than the animal portion, that being more resistant to such action than the lime, leaving us to conclude that the dissolved lime was in a more perfectly soluble condition than the former variety, and we might in consequence readily infer the action of either nitric, muriatic, or acetic acids in the latter case, and that of sulphuric in the former, the first three all being soluble salts, and the latter scarcely at all so. These inferences are not dicta, only hypothesis.

The white variety is more rapid and destructive than either of the others, being characterized by a softening of the whole substance of the dentine as far as the decay reaches, which easily chips away under the excavator, and is generally quite sensitive to the touch of the instrument, the line of demarkation not being readily determined by any one of limted experience, and in many instances not by the most expert and experienced, hence the result more doubtful and uncertain when filled. This variety is more especially confined to young subjects, not often met with in extreme old age. This latter or white variety does not slough away, as in the black, but remains in a more or less complete disorganized condition, not being removed *pari passu*, with the progress of the disintegration.

In the brown variety, the cartilage being retained, may be easily peeled up from the sound dentine below the cartilage so removed, showing under the microscope the tubular structure more or less intact, generally portions of them being corroded out from end to end, presenting a strongly marked baccated or beaded appearance, and I have entertained the idea that portions of such softened dentine, over the pulp cavity, in large decays, might be retained as a capping, far superior to any artificial capping, with a hope barely of subsequent ossification of this softened dentine, especially where there are live nerve fibrils, as we have reason to believe is sometimes the case where this softened portion remains sensitive to the touch at least, no harm could be apprehended from leaving a small portion where the borders of such cavities are sufficiently excavated.

I might extend these remarks, but for the fact that I like short articles, so as not to bore my readers.

Now in relation to the chemical formulas given in the article under consideration, there are some broad mistakes or errors in the nomenclature, perhaps from want of care in making them up or oversight, which I propose noticing in detail.

First formula: sulphuric acid, $H_2 S O_4$. As its formula indicates, it is 2 parts of hydrogen, by weight 32 of oxygen, 16 of sulphur. Now let us see how the facts stand. It will be seen by reference to any of the text-books on chemistry that sulphuric acid consists of $S O_3 + H O$; or sulphur, 16; oxygen, 24; and one equivalent of water, H O; or one part by atomic weight of hydrogen, and eight of oxygen, which is the hydrous acid; the unhydrous contains no water, and is a white solid, instead of a liquid form, $E_{40}$. The Nordhousen oil of vitriol consists of two atoms of acid and one of water, thus ($S\ O_3\ H\ O + S\ O_3$) equivalent to 89. This is the fuming sulphuric acid. It contains only half the amount of water, the equivalent of the common commercial acid being 49; the unhydrous being 40.

Let any person at all acquainted with chemical manipulation examine the formulas given for the formation of this acid in the mouth, he will at once come to the conclusion that it might be impossible for any such formation to take place, even if the necessary elements be present, the requisite conditions will be found wanting.

This acid is made on a large scale by burning sulphur with nitrate of potash or soda, and conducting the sulphurous and nitrous acids which result into large chambers lined with lead, in which steam is thrown, the bottom of the chamber being covered with water. The sulphurous acid takes oxygen from the nitrous acid, reducing it to deutoxide of nitrogen. This being in the presence of oxygen in the chamber, the deutoxide instantly reassumes the condition of nitrous acid. The deutoxide continually transfers oxygen from the atmospheric air to the sulphurous acid, and brings it to the condition of sulphuric acid. Thus it will at once be seen that this acid could not be found in the mouth, the requisite conditions being wanting, even if the elements be present.

The writer says if a fibre of meat be caught and retained between the teeth sufficient time, it decomposes part of its hydrogen uniting with part of the nitrogen, forming $N\ H_3$; its carbon uniting with part of the oxygen, forming carbonic acid, acid, $C\ O_2$; and the sulphur combining with the remaining hydrogen, forming sulphuretted hydrogen, $H_2\ S$. Now sulphuretted hydrogen in contact with oxygen of the air, is decomposed, the hydrogen uniting with the oxygen, forming water, and the sulphur set free. This sulphur being in the nascent state, and having a great affinity for oxygen, is oxidized, and the result is sulphurous acid, $S\ O_2$; which is converted rapidly into sulphuric acid, in the presence of the water of the saliva, thus: $S\ O_2 + 2\ H_2\ O\ H_2\ O_4\ S + 2\ H$. "The acid thus formed immediately acts on the carbonate of lime, decomposing it, and charring the animal portion." The formula for sulphuretted hydrogen is H. S., and not 2 atoms of hydrogen, as above given. This feeble acid gradually undergoes decomposition in water, forming water and sulphur set free, but does not readily undergo decomposition in the air, more especially in dry air. The equivalent of this acid is 17, instead of 18, as the above writer would express. The above formula for the formation of sulphuric acid from sulphuretted hydrogen will not do as he has there given, 3 parts of sulphur, 8 of oxygen, 10 of hydrogen, which is too high.

If it were possible for sulphuric acid to be formed at all out of hydro sulphurous acid, the formula would read thus: $S\ H\ H\ O\ O_3 = S\ O_2 + O\ H\ O$ Or in other words, a hydrated ter-oxide of sulphurous acid, making one part of sulphur, two of hydrogen, and four of oxygen, instead of 8 of oxygen, 3 of sulphur, and 10 of hydrogen, as given in the writer's formula above. I can not see how this acid could be possibly formed in the mouth at all.

Nitric acid symbol, $H\ N\ O_3$, as given by the writer, is not correctly stated, though corrected in the description below. Its symbol is $N\ O_5 + H\ O$, equal to 54 unhydrous, and as it always contains one equivalent of water, would be 9 equivalents more added, is the exact formula of the books. The author says: "It is commonly prepared by the action of sulphuric acid upon potassium nitrate; the reaction is as follows: $K\ O_3 N + H_2 O_4\ S = K\ H\ O_4 S + H\ N\ O_3$" None of these formulas are correctly given; he has given one equivalent of K, one of N, and one of S, which is correct, and two of H and 7 of O, which is incorrect, and does not contain enough, lacking three equivalents; neither are the groupings correct from beginning to end.

He further says it decomposes the carbonate of lime, setting the carbonic acid free, forming nitrate of lime and water, thus: $Ca\ O_3\ C + 2\ H\ N\ O_3 = Ca\ 2\ N\ O_3 + C\ O_2 + H_2\ O$ Now none of these formulas are correctly given; the correct formula is $Ca\ O + C\ O_2 + N\ O_5 + H\ O = Ca\ O + N\ O_5 + H\ O\ O_2$. It will be seen that the author has given one equivalent too much of hydrogen. He states that the acid decomposes the carbonate of lime and dissolves out the phosphate of lime, but can not decompose it, and destroys the animal portion. I will advert to this further on. A piece of meat between the teeth, undergoing decomposition, might set free the elements to form nitric acid; but we have no proof of its formation in the mouth in this or any other way, and I doubt the possibility.

Hydrochloric acid symbol, $H\ Cl$. He says this acts energetically on the teeth. This is certainly true, as this is a very destructive agent when in contact with the teeth, either in or out of the mouth. His formula is not correct in point of atoms of equivalents, and not put together in the most intelligent manner, thus: $Ca\ O_3\ C + 2\ H\ Cl = Ca\ Cl_2 + H_2\ O\ C\ O$. The more accurate statement would be thus: $Ca\ O\ C\ O_2 + H\ Cl = Ca\ Cl + C\ O_2 + H\ O$; that is, the oxygen of the oxyide of lime in union with carbonic acid would be replaced by chlorine, and the carbonic acid gas and water formed and set free. The atom of oxygen of the lime is liberated, and at the same time the hydrochloric acid being decomposed, its hydrogen uniting with the liberated oxygen, forming water and chloride of lime, carbonic acid escapes as gas.

The error in the writer's formula is in giving 2 atoms of hydrochloric acid instead of one, and also the resultant bichloride of lime instead of protochloride. He has not given oxygen enough to unite with his carbon and hydrogen to form two atoms of water and one of carbonic acid.

Chemical nomenclature is exact and definite in its terms, every letter and figure having a specific value under all circumstances.

Now the writer maintains that none of the three strongest of acids has any power to decompose the phosphate of lime in the tooth substance, and that only the carbonate is decomposed, and the phosphate dissolved, notwithstanding the carbonate forms a small per cent. of the tooth substance, only 5⅓ part in the hundred. We have no facts to prove that the two salts of lime in the tooth composition are themselves chemically united before or after entering into tooth composition; if so, even perhaps the removal of the carbonic acid, if such could be possible, would not cause the phosphate to dissolve out in the presence of a weak acid, reactions such as may and do exist in the mouth, causing the destruction of teeth.

What are the facts in the case. It is known to analytical chemists that each and all of the acids spoken of are capable of acting on tooth substance and decomposing the phosphate of lime as well as the carbonate. The phosphate of lime found in the bones and teeth is a tribasic or neutral salt of lime; that is, three equivalents of lime unite with one equivalent of phosphoric acid, which forms an insoluble salt of lime, some times called basic salt.

Now when sulphuric acid is added to teeth or bones, the acid having a strong affinity for lime, one equivalent of the acids unites with one equivalent of the tribasic phosphate of lime, forming an equivalent of sulphate of lime, leaving two equivalents of lime in union with the phosphoric acid, forming a bibasic phosphate, which is now an acid salt and also a soluble salt, which is readily dissolved away by water or almost any other fluid; at the same time the carbonate of lime is converted into a sulphate, not at first in a crystalline condition. What has been said of sulphuric may be said of nitric, muriatic, and even other acids. They may all be supposed to act in the same way, which is in fact already proven by experiments on bones, in the manufacture of artificial manures, besides various other experiments by the ablest chemists.

All of the three mineral acids have strong affinities for water, and readily remove it from all organic tissues, more actively so when concentrated, leaving the carbon behind and the organic structure broken up and disorganized, so far as the acid action extends into the substance, readily removing the water or its elements from corks in bottles containing any of the mineral acids, leaving the organic tissue broken up with the carbon—left behind in a disorganized condition, which when the acid is neutralized and washed out and dried, burns without flame.

The corks or wood so acted on by the different acids are not all the same color; that of sulphuric, dark; that of nitric, light color; the muriatic is also light, but of a different shade, which fact may have left the writer of the article alluded to to the conclusion that the different forms of decay mentioned were the result of the action of the different acids. This fact is not well established, as we some times find more than one form of decay in the same mouth at the same time.

The mineral acids undiluted have scarcely any action on metals, owing to the fact that the normal amount of water contained in the undiluted acid, which the acid will not give up readily to the metal to form the requisite oxide, from which the salt is formed free, water must be present, held by the acid by a weak affinity, the water readily uniting with the metal to form an oxide, then the free acid unites with the oxide, atom by atom, to form the salt of the metal.

